# Complexed Polymer Film-Forming Spray: An Optimal Delivery System for Secretome of Mesenchymal Stem Cell as Diabetic Wound Dressing?

**DOI:** 10.3390/ph15070867

**Published:** 2022-07-14

**Authors:** Abd. Kakhar Umar, Jittima Amie Luckanagul, James H. Zothantluanga, Sriwidodo Sriwidodo

**Affiliations:** 1 Department of Pharmaceutics and Pharmaceutical Technology, Faculty of Pharmacy, Universitas Padjadjaran, Sumedang 45363, Indonesia; 2 Department of Pharmaceutics and Industrial Pharmacy, Faculty of Pharmaceutical Sciences, Chulalongkorn University, Bangkok 10330, Thailand; jittima.luck@gmail.com; 3 Department of Pharmaceutical Sciences, Faculty of Science and Engineering, Dibrugarh University, Dibrugarh 786004, Assam, India; jameshztta@gmail.com

**Keywords:** stem cell’s secretome, regenerative therapy, diabetic wound healing, carboxymethyl chitosan, hyaluronic acid, collagen tripeptides, film-forming spray

## Abstract

Diabetes-related wounds have physiological factors that make healing more complicated. High sugar levels can increase microbial infection risk while limiting nutrition and oxygen transfer to the wound area. The secretome of mesenchymal stem cells has been widely known for its efficacy in regenerative therapy. However, applying the secretome directly to the wound can reduce its effectiveness. In this review, we examined the literature on synthesizing the combinations of carboxymethyl chitosan, hyaluronic acid, and collagen tripeptides, as well as the possibility of physicochemical properties enhancement of the hydrogel matrix, which could potentially be used as an optimal delivery system of stem cell’s secretome for diabetic wound healing.

## 1. Introduction

Diabetes mellitus is a global illness with substantial morbidity and mortality. According to WHO data, the number of diabetics is expected to reach 693 million by 2025 and rise further. Diabetic wounds have the greatest morbidity rate of any diabetes complication. Diabetic lesions may generally be cured (60–80% of the cases), but 10–15% remain active, and 5–24%require amputation within 6–18 months. Neuropathic wounds often heal in more than 20 weeks, whereas neuroischaemic injuries take longer and frequently end in amputation [1]. This is supposed to be addressed by regenerative treatment and wound dressings.

Treatment using mesenchymal stem cells (MSCs) for regenerative therapy, implantation, and protein supply for wound healing is becoming more widespread [2,3,4,5,6,7]. However, there are numerous drawbacks to using MSCs. MSCs cannot be grown and stored for extended periods [8,9]. Immune system resistance, tumor or cancer growth, atherogenesis, and arrhythmogenesis can occur [10,11]. Recent research also implies that the therapeutic impact is caused by the release of paracrine agents such as cytokines, growth factors, and exosomes rather than stem cell transdifferentiation and engraftment. These biomolecules are known as the secretome, which plays a critical role in communication between cells. Therapy using secretome is better than cell-based therapy [12,13]. Secretomes can restore diabetic and corneal injuries without leaving scars [14,15,16]. It can also be produced in larger numbers and preserved for a longer period of time than stem cells [16,17]. As such, there has been developing intrigue within the use of secretome within the clinical field, mainly because it has a few focal points over the conventional utilize of stem cells in regenerative pharmaceutical treatment, counting expanded ease of conveyance, decreased concerns for oncogenic potential related with stem cell utilize, need of immunogenic response empowering allogeneic or off-the-shelf utilization, and vast potential for in vitro modulation [6,12].

Direct administration of the secretome may reduce its effectiveness. Since the secretome depletes quickly owing to enzymatic destruction or migrates to other organs, it is frequently administered in high quantities or repeated doses [18]. Large doses can result in dose-dependent cytotoxicity [19]. Within 30 min of injection, the secretome can extend to other tissues/organs such as the lung, liver, kidney, spleen, muscle, heart, and possibly the brain [20,21]. As a result, to optimize the secretome’s retention and potency in the target tissue, a controlled and localized delivery system is required. Several natural polymers are reliable as multi-biomolecular delivery systems with bioadhesive and biocompatible properties such as chitosan, collagen, hyaluronic acid (HA), and their combinations [22,23]. The combination of chitosan, collagen, and HA hydrogel can form a biomimetic environment that supports accelerated cell proliferation, differentiation, and colonization while stimulating wound angiogenesis [24,25]. Chitosan is a natural antioxidant, antimicrobial, and antitumor [26,27,28], while collagen and HA are produced naturally by the body and play an essential role in wound healing [29,30]. These hydrogels also have beneficial properties in localized delivery, namely in situ film or viscoelastic properties, so they can be administered directly by injection or spray into the target tissue [25,31].

**Figure 1:**
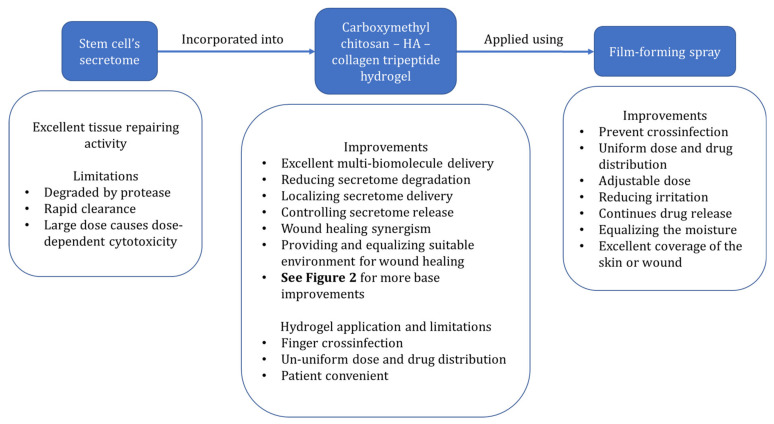
The framework of thinking.

Drug delivery using the film-forming spray (FFS) has several benefits over traditional topical treatments, including homogenous drug distribution and dose, higher bioavailability, decreased risk of irritation, sustained drug release, and faster wound healing via moisture control [31]. Cross infection from the finger to the wound area commonly occurs in conventional preparations and can be prevented using FFS. Therefore, in this opinion, we discussed a potential controlled and localized secretome delivery system using chitosan-HA-collagen complex hydrogel in the film-forming spray for chronic diabetic wounds. The framework of thinking can be seen in Figure 1.

## 2. Diabetic Ulcer

Diabetic wounds are one of the complications of diabetes with the highest morbidity rate. Generally (60–80%), diabetic wounds can be healed, whereas 10–15% will remain active, and 5–24% of them end up with amputation in 6–18 months. Neuropathic wounds generally heal in more than 20 weeks, whereas neuroischaemic injuries take longer and frequently result in amputation [1].

Risk factors for diabetic ulcers are diabetic neuropathy, peripheral arterial disease, and foot trauma. Neuropathy is a common factor in nearly 90% of diabetic wounds. Diabetes causes nerve damage to the motor, sensory, and autonomic fibers. Muscle weakness, atrophy, and paresis are all symptoms of motor neuropathy. Sensory neuropathy is characterized by pressure loss, pain, and heat sensitivity. This loss of sensitivity can result in repeated injury without realizing it. Autonomic dysfunction resulted in vasodilation and lessened sweating [32], resulting in skin integrity loss and an environment susceptible to microbial infection [33].

Meanwhile, the peripheral arterial disease causes nutrients and oxygen to the wound area to decrease so that the basic needs of cells for proliferation and differentiation and the availability of the immune system as protection are not met. For these reasons, treatment of diabetic wounds should target one of the following: (1) Keeps skin integrity intact through moisture control in the wound area [34]; (2) Preventing increased severity by microbial infection [35]; (3) Meeting the needs of nutrients, paracrine factor, and oxygen to the wound area through repair of the arterial system, the formation of new blood vessels, or direct supply to the wound area [36,37]. Regenerative therapy and the use of wound dressings are said to address all three of these problems.

## 3. Application of Mesenchymal Stem Cells and Secretomes in Diabetic Wound Healing and Their Limitations

Stem cell therapy has recently emerged as an innovative intervention strategy for treating diabetic wounds. The types of stem cells that are most often used are adult stem cells such as bone marrow-derived mesenchymal stem cells (BM-MSC), adipose-derived stem cells (ADSC), human umbilical cord-derived mesenchymal stem cells (hUC-MSC), and peripherals blood-derived mesenchymal stem cells (PB-MSC) [36]. Cytokine and chemokine receptors’ expression supports the migration and integration of MSCs on the cell surface. The chemokine receptor-ligand interaction (CXC 4, CC type 2 cytokine receptor, CCR7, integrin α4, and integrin β1 comes into contact with vascular cell adhesion protein 1 on endothelial cells) is functionally involved in MSCs homing [37,38,39]. MSCs secrete matrix metalloproteinase 2 (MMP-2) to transmigrate across the single layer of the endothelium to accelerate the laying process [40,41,42]. MSCs have been shown to contribute to increased vascular density and restoration of sensory function by secreting keratinocyte growth factor (KGF), VEGF, and platelet-derived growth factor (PDGF) [41,43]. More importantly, MSCs have also shown significant therapeutic potential for reversing diabetic femoral nerve degeneration (FN) by increasing the capillaries in the FN-supplied gastrocnemius, expression of nerve growth factor (NGF), and restoring FN slow conduction in a diabetic-induced rat wound model [44]. However, MSCs applications have several limitations that need to be considered.

MSCs are not stored and do not remain in the wound tissue for a long time. Usually, it fades away over 24 h since it does not adhere well and migrates to other tissues [45]. The limitations of standardization and optimization criteria are challenges in using MSCs. There are many variations in the application of MSCs, including the heterogeneity of donor-based MSCs, isolation and cell culture conditions, cryopreservation methods, dosage, frequency, injection route, cell administration time point, and follow-up period. In addition, MSCs have the potential to produce untargeted tissue differentiation, undesired immune responses, tumorigenicity, and, most importantly, malignant promotion and transformation [46]. Studies have also demonstrated that the therapeutic effect is caused by the release of paracrine factors such as growth factors, cytokines, and exosomes rather than MSCs transdifferentiation and engraftment [12,13,47,48,49,50,51,52]. This issue drives regenerative research focused on the use of the secretome.

The secretome contains a variety of paracrine factors and other biomolecules that play an essential role in cell communication. The direct supply of paracrine factors will be handy to meet the material needs in the granulating tissue formation, re-epithelialization, angiogenesis, and collagen metabolism [46]. Secretome in a conditioned medium has been shown to increase epidermal growth factor (EGF) and basic fibroblast growth factor (bFGF) gene expression in the wound area so that it improves fibroblasts behavior [53]. Secretome also increases eNOS-specific mRNA (Nos3) levels and supports the restoration of the SDF1/CXCR4 axis in diabetic EPCs [54,55], which leads to increased keratinocyte proliferation [56]. C. D. Gregorio et al. (2020) reported that secretome increased thermal and mechanical sensitivity, restored intraepidermal nerve fiber density, reduced neuron and Schwann cell apoptosis, increased angiogenesis, and reduced peripheral nerve inflammation in diabetes-induced wounds [57].

Clinical trials using secretome in treating persistent corneal epithelial defects (PEDs) in various etiologies reveal promising outcomes. There was a substantial decrease in the mean PED area after 28 days of therapy compared to baseline (66.4 ± 35.3%, *p* = 0.001). Five eyes (41.7%) obtained full wound closure after 28 days of therapy. There were no serious adverse events associated with the medication [58]. The administration of apoptotic peripheral blood mononuclear cell secretome (PBMCs) to artificial wounds, on the other hand, did not affect wound closure but was safe and well-tolerated by human skin (a randomized phase 1 experiment). Two other clinical trials using the same approach failed to demonstrate secretome’s efficacy on diabetic wound healing [59]. This might be due to the secretome’s incompatibility with the patient and in vivo instability after administration.

The secretome’s effectiveness depends on the target cell endocytosis and close connection with the cell surface receptors, which necessitate the secretome’s presence at the target site for an extended period [60]. Administration of secretome without a delivery system will not last long in the target tissue, or its effectiveness will decrease due to enzymatic metabolism or seeping into other tissues [20,21]. Natural polymers can be used as safe, controlled, and localized delivery of biomolecules, given their biodegradable, biocompatible, and non-immunogenic properties [61].

## 4. Complexation Possibility of Chitosan-Collagen-Hyaluronic Acid

Chitosan is a natural polymer with antitumor, antioxidant, and antimicrobial effects. These properties are very useful in inhibiting the growth of microbes in the diabetic wound area. With the ability to absorb and donate moisture in the wound area, chitosan can maintain fluid balance in diabetic wounds to form a physiological environment suitable for wound healing [62]. Apart from its role as a wound dressing, chitosan can be used as a delivery matrix with a continuous release [63]. Chitosan can form matrix films in situ under environmental conditions with pH > 6.5 [64]. When the pH increases, chitosan undergoes deionization and produces a three-dimensional network [65].

Protecting its contents, adhering to mucosal surfaces, and opening tight junctions between epithelial cells are three unique properties that make chitosan a suitable polymer for delivering proteins and peptides via different administration routes. Various forms of derivatives and complexation with other polymers can increase the solubility, biodegradability, mucoadhesive, and transfection efficiency of chitosan [23]. One of the derivatives of chitosan is carboxymethyl chitosan which has better solubility in water. Carboxymethyl chitosan (CC) has better bacteriostatic properties and dressings and is the potential to be used as biomolecule delivery with a sustainable release [66]. By complexing with other polymers such as hyaluronic acid (HA), the system successfully delivers genes and increases their cell internalization through interactions between hyaluronic acid and CD44 receptors. HA-chitosan nanoparticles have high transfection levels without affecting cell viability [23]. Complexation of carboxymethyl chitosan—hyaluronic acid (CCHA) can be achieved by forming cross-amide bonds and modified aldehyde groups from HA, forming a porous structure on the hydrogel. The porous hydrogel has better wound dressing properties with substantial cellular infiltration and sufficient ECM deposition. Cellular and cytokine responses increase in inducing angiogenesis after the administration of CCHA hydrogel. An adequate blood supply will significantly improve the tissue regeneration process so that the system is suitable for use as diabetic wound dressings. The in situ film-forming CCHA hydrogels can be produced via Schiff’s base reaction [25].

**Figure 2:**
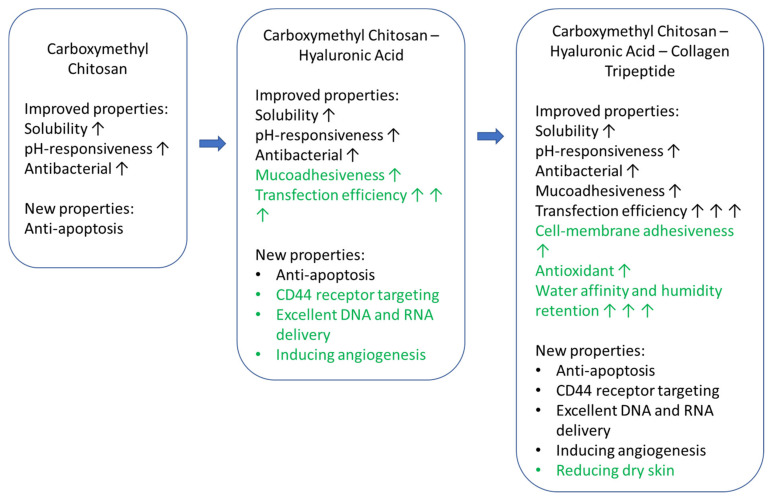
The potential improved and new properties of carboxymethyl chitosan—hyaluronic acid—collagen tripeptide complex. Note: ↑ for enhanced properties and ↑↑↑ significantly improved properties.

To increase the secretome retention time on the cell surface, tripeptide from collagen can be used in complex chitosan. Collagen tripeptides (CTP) have good biocompatibility and the ability to support cell proliferation and adhesions [67]. CTP itself is a hydrolyzed form of collagen, so, with a lower molecular weight, it is more easily absorbed. CTP comprises three peptides with a Gly-X-Y sequence (e.g., Gly-Pro-Hyp). Carboxymethyl chitosan—collagen tripeptide (CCCTP) has a significantly better water affinity, moisture retention, and antioxidant capacity than collagen. The elasticity of the CCCTP film is also excellent making it suitable for clinical applications. The elastic matrix is said to support the proliferation and migration of fibroblast cells significantly compared to collagen alone as a control [68,69]. The potential improved and new properties of the CCHACTP complex can be seen in Figure 2.

**Figure 3:**
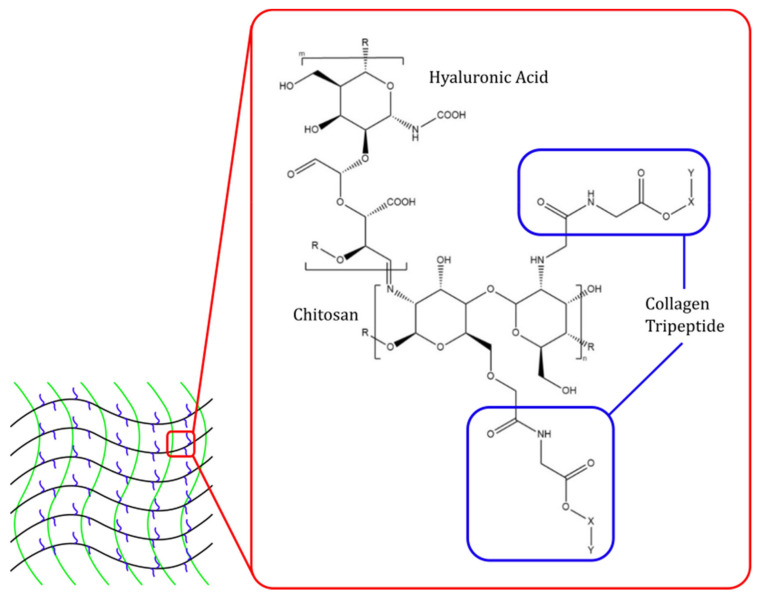
Structure and matrix illustration of carboxymethyl chitosan—hyaluronic acid—collagen tripeptide (CCHACTP) complex.

CCCTP complex hydrogel can be obtained by quaternizing the carboxymethyl chitosan using 2,3-epoxypropyl trimethylammonium chloride, and then the mixture is dialyzed and lyophilized to obtain yellow powder (QCC). Next, N-hydroxy sulfosuccinimide (NHS) and 1-ethyl-(dimethyl aminopropyl) carbodiimide (EDC) are reacted with QCC to activate the COOH group. After the activation, CTP is added to the solution to form the CCCTP complex [69]. The structure and matrix illustration of the carboxymethyl chitosan–hyaluronic acid–collagen tripeptide (CCHACTP) complex can be seen in Figure 3.

## 5. The Use of Chitosan, Hyaluronic Acid, Collagen, and Their Combination in Secretome Delivery

The study by Saberpour, M., et al. (2019) showed that the conditioned medium of MSCs can be loaded into chitosan nanoparticles (MC-CM-CS NPs) and form crosslinking. The adsorption efficiency was up to 77%, with a particle size of ~414 nm. The release of the conditioned medium from chitosan nanoparticles reached 72% after 72 h at pH 7.2. The highest decrease in Toll-like receptor 4 (TLR4) expression was obtained from the MC-CM-CS NPs system with a percentage of 90%. This was higher than the conditioned medium from MSCs and chitosan itself, with a decreased TLR4 expression percentage of 89% and 82%, respectively. This shows that the antibacterial effectiveness of secretome can be increased through incorporation into chitosan nanoparticles [70]. The polymerization of chitosan and MSCs’ exosomes can be carried out at low temperatures (−20 to 4 °C) to prevent the destruction of exosomes. It was found that there was no significant difference in micro-and macrostructure between the chitosan hydrogel polymerized at high and low temperatures. This low-temperature polymerized chitosan system can load as many as 183.08 ± 15.44 × 10^8^ exosomes. The release of exosome particles could also be slowed down to ~80 × 10^8^ particles on day 6. The proliferative activity of fibroblast cells and angiogenesis of exosomes became significantly higher by controlling their release using chitosan hydrogel [71].

Three-dimensional gels of collagen have been widely used as a medium for MSCs and play an important role in regulating secretome release. The total protein content in the collagen gel was maintained and released slowly (time-dependent) up to day 28 (~270 to 6 ng). This regenerative protein cargo’s delivery increases endogenous cells’ regeneration capacity [72]. Clinical studies have also shown that delivery of MSCs and their secretomes do not cause side effects. This confirms that collagen as multiple biomolecular carriers is safe and feasible [73]. Collagen hydrogel containing secretome also reduces the proliferation of allogeneic lymphocytes, lowering the likelihood of tumor formation, especially at high collagen concentrations [74]. Other literature reports that secretome is more stable, and its therapeutic effect is increased using a collagen base [75,76]. Furthermore, in third-degree burns, the combination of collagen and chitosan thermosensitive hydrogel containing secretome was able to reduce inflammation, stimulate the creation of granulation tissue with high re-vascularization, and prevent the formation of hypertrophic scar [77]. Collagen-chitosan microbeads stimulate the expression of osterix, osteocalcin, and calcium mineral deposition, resulting in the optimum impact on orthopaedic tissue treatment [78]. β-glycerophosphate is often used as a crosslinker for complexing these systems [77,78].

Integrating crosslinked hyaluronic acid can also help to stabilize the secretome. Secretome release occurs concurrently with the biodegradation of crosslinked HA, resulting in continuous release [79]. The HA spongy hydrogel can limit the number of macrophages in the wound region, allowing the secretome to remain stable in vivo [80]. The synergistic effect of secretome and HA viscoelastic gel was also reported by Rogers, GFC. et al. (2018), where the administration of HA and secretome alone had a significantly lower proliferative effect than the combination [81]. Furthermore, miR21-loaded crosslinked chitosan-HA nanoparticles could enhance osteogenic differentiation of cell sheets and increase the expression of calcifying genes, collagen levels, and mineral deposits [82].

The secretome can be loaded into CCHACTP concurrently with the complexation of chitosan and HA, allowing the secretome to be adsorbed before the complex forms a net as a physical barrier. This can be carried out at low temperatures to avoid denaturation of the secretome components [71]. The presence of collagen tripeptides in each layer of the chitosan-HA matrix allows for forming physical and chemical interactions with secretome proteins, improving absorption efficiency. Furthermore, because the hydrogel component is made of natural materials and is biodegradable, the secretome’s release will coincide with the breakdown of the CCHACTP matrix’s outer layer. This will provide a time-dependent pattern on the secretome’s delayed release, providing levels of the secretome in line with the therapy.

## 6. Conclusions

Treating diabetic ulcers can be more efficient by maintaining epidermal integrity, minimizing infection, and enhancing nutrition and oxygen delivery to wound tissue. Since it can address these needs, MSCs’ secretome meets the criteria for an excellent regenerative therapy for diabetic wounds. The therapeutic impact of secretome can be considerably boosted through delivery system adjustment and synergism. Based on the data we have collected, in our opinion, a film-forming spray of carboxymethyl chitosan–hyaluronic acid–collagen tripeptide (CCHACTP) hydrogel matrix is the most optimal delivery system for MSCs’ secretome as a diabetic wound dressing.

## Data Availability

Data sharing not applicable.
